# Effects of Screw-Press Temperatures on Oil Yield and Phytoconstituents of Roasted and Unroasted Black and White Sesame Cultivars

**DOI:** 10.3390/molecules31101639

**Published:** 2026-05-13

**Authors:** Parbat Raj Thani, Chamini Kaushalya Welhena, Mani Naiker, Tieneke Trotter

**Affiliations:** School of Health, Medical and Applied Sciences, Central Queensland University, Rockhampton, QLD 4701, Australia; c.welhena@cqumail.com (C.K.W.); m.naiker@cqu.edu.au (M.N.); t.trotter@cqu.edu.au (T.T.)

**Keywords:** sesame seeds, screw-press extraction, effect of temperature, oil yield, TPC, antioxidant capacity, fatty acids, sesamin, sesamolin, sesamol

## Abstract

In the context of exploring new methods and techniques for the sustainable production of high-quality oil, the present study aimed to evaluate the influence of temperatures during screw-press extraction and roasting (200 °C for 15 min) on the oil yield, phytochemicals, and antioxidant contents of white and black seeds of *Sesamum indicum* L. In this study, the oil of roasted and unroasted white and black sesame seeds was extracted using the screw-pressing technique at various temperature increments between 40 and 200 °C. The total phenolic contents (TPC), antioxidant capacity (FRAP (Ferric Reducing Antioxidant Capacity) and CUPRAC (Cupric Reducing Antioxidant Capacity)), lignans (sesamin, sesamolin, and sesamol contents), and fatty acids were quantitatively determined by spectrophotometry, high-performance liquid chromatography, and gas chromatography–mass spectrometry. The results revealed that the values of TPC, antioxidant capacity and sesamol in black seeds or its derived oil were significantly greater than those present in equal quantities of white seeds or its derived oil; however, oil yield, sesamin, and sesamolin were higher in white sesame. When temperatures increased up to 150 °C during seed pressing, this resulted in increased oil yields of unroasted seeds; however at 200 °C, the oil yield dropped slightly. The roasted seeds resulted in slightly less oil yield than unroasted seeds, which could be attributed to seed moisture. Moreover, sesamin, sesamolin, sesamol, and fatty acid compositions of unroasted and roasted seeds did not show considerable changes with increased temperatures during seed pressing. In fact, these phytoconstituents were found to possess robust screw-press thermal stability, while TPC and antioxidant capacity showed considerably negative effect with increasing screw-press temperature. The results highlight that moderate pressing temperatures optimize oil yield while maintaining the integrity of key bioactive constituents, emphasizing the potential of sustainable mechanical extraction for high-quality sesame oil production.

## 1. Introduction

Sesame (*Sesamum indicum* L.), a member of the Pedaliaceae family, is a well-known crop valued for its nutrient rich seeds. The seeds contain dietary fiber, carbohydrates, proteins, fats, minerals, and vitamins, all of which directly contribute to their nutritional value [1,2]. They are also a valuable source of health-benefiting compounds such as lignans (particularly sesamin, sesamolin, and sesamol), fatty acids, sterols, and tocopherols, which are recognized for their therapeutic properties [2,3,4].

Among the various phytoconstituents found in sesame seeds, lignans (sesamin, sesamolin, and sesamol) and unsaturated fatty acids are the key compounds that make sesame highly valued. These components are predominantly present in seed oil and are largely responsible for its nutritional and functional importance. Owing to this phytochemical composition, sesame oil has extensive applications in the food and health industry. In food, sesame is used in cooking, condiments, and confectionery products, where it adds a distinctive nutty flavor [5]. Medicinally, sesame exhibits antioxidant and anti-inflammatory effects that may help manage conditions such as atherosclerosis, diabetes, and hypertension [2]. Industrially, it is used in pharmaceuticals, cosmetics, lubricants, and even in emerging sectors such as biodiesel production [6].

Several oil extraction techniques are available for sesame seeds, including traditional techniques such as cold and hot mechanical pressing, solvent-based extraction (supercritical CO_2_ extraction, Soxhlet extraction, reflux extraction, ultrasound-assisted extraction, and microwave extraction), and enzyme-assisted extraction [7,8,9,10]. Previous studies have shown that both oil yield and key phytoconstituents concentration, such as lignans and fatty acids vary considerably in sesame oil, depending on the employed extraction method [11,12,13,14]. These variations can notably affect the sustainability of the oil production and ultimately determine the nutraceutical and pharmaceutical value of the final product [15].

Among all available techniques, mechanical pressing remains the most widely practiced and industrially preferred extraction method, largely due to its well-recognized advantages such as low operational cost, simple equipment requirements, absence of solvent residues, high-quality oil output, and production of oil suitable for direct consumption [16,17,18]. Despite these advantages, numerous studies, including Fasuan, et al. [19], report that mechanical extraction generally produces lower oil yields compared with solvent-based or supercritical methods, which can reduce overall processing efficiency and profitability. Furthermore, even within mechanical pressing systems, several operational variables, including seed moisture content, pressing temperature, screw rotation speed, and feeding rate, have been shown to significantly affect both oil yield and the retention of important phytoconstituents across various oilseed species [10,16]. Therefore, optimizing these processing parameters is crucial to achieving efficient, high-quality, and sustainable oil production.

Among these variables, temperature plays a particularly critical role, influencing not only the flow properties of the oil but also the thermal stability of valuable phytoconstituents. However, despite its importance, there remains limited information on how pressing temperature directly affects sesame oil yield and composition. Hence, the aim of this study was to explore the relationship between pressing temperature and the qualitative and quantitative attributes of unroasted and roasted black and white sesame seed oil, and to identify the optimal extraction temperature that maximizes both yield and quality.

## 2. Results and Discussion

### 2.1. Total Extractable Oil Content

The Soxhlet extraction method with *n*-hexane was used to obtain the total extractable oil yield (total lipid content) from fresh white and black sesame seeds (Table 1).

The white and black sesame seeds had total extractable oil yields of 50.9% and 48.1%, respectively (Table 1). Since many researchers have extracted oil from sesame seeds using similar methods applied in the present study and reported total extractable oil yields in the range of 20.7–58.2% from across the world, the results obtained in the present study suggest that the seeds used were relatively rich in oil [20,21,22].

Furthermore, white sesame exhibited slightly higher extractable oil content than black sesame. Several previous studies have reported similar results [20,22,23,24]. Chakraborty et al. [23] reported the highest extractable oil yield, approximately 46%, in white sesame, and the lowest extractable oil yield, approximately 33%, in black sesame from Bangladesh. Sani et al. [20] reported 20.7% and 47.8% extractable oil yields in brown and white seeds, respectively, in Nigeria. Agidew et al. [22] reported white sesame seeds containing 53.2–58.2% extractable oil versus 47.6–51.4% in black varieties from Ethiopia. Nurhayati [24] also supported this trend, noting white sesame’s extractable oil content at 37% compared to 35.5% in black sesame in Indonesia. These comparisons support the conclusion that white sesame tends to contain slightly more extractable oil than black sesame, although the magnitude of the difference varies by region and cultivar.

### 2.2. Screw-Pressed Temperatures and the Product and Byproducts of Fresh and Roasted White and Black Sesame Seeds

#### 2.2.1. Fresh White and Black Sesame Seeds

The oil yield and solid residue (sludge and seedcake) were obtained from fresh white and black sesame seeds extracted at various screw-pressed temperatures starting from 40 °C to 200 °C (Table 2).

It is evident that the screw-press oil yields were much lower than the total extractable oil (Table 2). White sesame produced 18.5 to 36.8% oil through screw-pressing, compared with a total extractable oil yield of 50.9%, meaning only about 36 to 72% of the available oil was recovered. Black sesame showed a similar pattern, with screw-press yields of 11.6 to 32.6% against a total extractable oil yield of 48.1%, corresponding to roughly 24 to 68% recovery. This highlights the limited extraction efficiency of mechanical pressing compared with the total oil present in the seeds.

The results also showed a consistent increase in oil yield with rising screw-press temperature up to 150 °C for both fresh white and black sesame seeds. Alongside the increase in oil yield, both sludge and seedcake percentages decreased with temperature, suggesting more complete oil expulsion and more complete cell wall rupture at higher processing temperatures. This trend aligns with previous studies on different oilseeds [10,16,25,26,27]. For example, Piravi-Vanak et al. [10] investigated the oil yield of two sesame varieties, Darab and Tak Shakhe Naz, at four screw-press temperatures (30, 60, 90, and 120 °C). They found that oil yield increased from 38% to 48% in the Darab variety and from 42% to 51% in the Tak Shakhe Naz variety as the pressing temperature increased. These results support the idea that higher pressing temperatures reduce oil viscosity and enhance cell wall rupture, thereby promoting smoother oil flow and recovery during mechanical extraction of various oilseeds.

However, oil yield declined slightly beyond 150 °C. The oil yield of fresh white and black sesame was reported 36.6% and 31.1%, respectively, at a 200 °C screw-press temperature, indicating that excessively high temperatures may reduce extraction efficiency. Many researchers have reported this phenomenon while examining different oil seeds [27,28]. For example, Allay et al. [27] investigated hemp seed oil yield under different screw-press temperatures (60, 80, 100, 120, and 140 °C) and reported that the oil yield increased up to 100 °C, but didn’t continue to rise beyond that point. This might be due to many reasons, including the effect of temperature on protein-lipid interaction [29].

#### 2.2.2. Roasted White and Black Sesame Seeds

These white and black sesame seeds, which were roasted at 200 °C for 15 min, were screw-pressed at 40 °C and 200 °C to evaluate the effect of roasting temperature on oil yield and solid residue yield (Table 3). The results show that the oil yield increased with rising screw-press temperature for both roasted white and black sesame seeds. The oil yield of white sesame increased from 12.7% at 40 °C to a maximum of 32.5% at 200 °C, while black sesame increased from 7.7% to 27.9% over the same temperature range.

However, roasting reduced oil yield when the results from unroasted and roasted sesame seeds were compared (Table 2 and Table 3). It is also noticeable in Table 2 and Table 3 that roasted seeds consistently produced lower oil yield than unroasted seeds at both 40 °C and 200 °C. Roasting lowered the yield by about 25% at 40 °C and 10% at 200 °C in white sesame. The reduction was about 20% at 40 °C and 9% at 200 °C for black sesame. This finding contrasts with most previous studies, which generally reported higher oil yields in roasted sesame seeds than in the unroasted sesame seeds [30,31,32]. For instance, Arab et al. [30] observed an increase in oil yield from 33.5% in unroasted seeds to 62.6% at 250 °C, followed by a decline in yield (56.3%) at 300 °C. Similarly, El Hanafi et al. [31] examined 16 roasting conditions (130–160 °C for 40–120 min) and found that oil yield increased up to 140 °C, then decreased at higher roasting temperatures. Their findings indicate that although excessive roasting temperature and duration can reduce oil yield, the oil content in roasted seeds typically remains higher than that of unroasted seeds under comparable conditions to those used in the present study. The contradictory results in the present study could be due to several factors. It is important to note that all those comparisons in oil yield of roasted and unroasted seeds in the previous studies were based on the oil yield obtained from the solvent extraction method, where oil is removed through dissolution, not the mechanical extraction method. Since the mechanical nature of pressing (pressing temperature and pressure, feeding rate, nozzle size, shaft screw diameter, rotational speed, and restriction size) and seed characteristics (moisture content, size, density, porosity, and frictional properties) may influence oil recovery [16,29,33]. For example, roasting drastically reduces the moisture content in seeds. In this study, unroasted seeds contained about 6% moisture, while roasting at 200 °C for 15 min likely reduced the moisture level to below 2%. According to previous reports, both higher moisture content and excessive dryness can lower screw-pressed oil yield, since an optimal moisture level is necessary to soften the seed structure and facilitate oil release [34,35,36]. Therefore, the very low moisture content in roasted seeds might have led to reduced oil yield compared to unroasted seeds in screw-press extraction. This study suggests reconstituting the moisture level of roasted sesame seeds at its optimum level before expelling.

### 2.3. Phytochemistry of Fresh and Roasted White and Black Sesame

#### 2.3.1. Fresh White and Black Sesame Seeds

The total phenolic content (TPC), antioxidant capacity (Ferric Reducing Antioxidant Capacity (FRAP) and Cupric Reducing Antioxidant Capacity (CUPRAC)), and lignan composition (sesamin, sesamolin, and sesamol) in fresh white and black sesame seeds were assessed (Table 4). The data clearly show that black sesame seeds contained significantly higher levels of TPC (126.7 mg gallic acid equivalents (GAE)/100 g dry weight (DW)) compared to white sesame seeds (106.6 mg GAE/100 g DW). Similarly, the antioxidant capacity measured by both FRAP and CUPRAC assays was greater in black seeds (159.8 mg Trolox equivalents (TE)/100 g DW and 733.9 mg TE/100 g DW, respectively) than in white seeds (138.9 mg TE/100 g DW and 428.3 mg TE/100 g DW, respectively). The results obtained in the present study are coherent with the many previous studies, including Shahidi et al. [37], who have reported a higher TPC value in black sesame seeds (29.9 mg catechin equivalent/g) and a lower TPC value in white sesame (10.6 mg catechin equivalent/g). In contrast, some previous studies have reported opposite results compared to the results obtained in the present study. For example, Agidew et al. [22] investigated TPC in the black and white sesame seeds grown at three different locations in Ethiopia and reported comparatively higher TPC values in white sesame (4.59–6.96 mg GAE/g) than in black sesame seed samples (2.95–4.27 mg GAE/g). These discrepancies indicate that black sesame does not always exhibit superior TPC or antioxidant capacity. Instead, different agro-climatic conditions, such as soil composition, climate, and seed maturity, are likely to interact to influence these bioactive compounds. Therefore, while black sesame seeds in this study demonstrated greater antioxidant potential, these results should be interpreted considering the influence of other contributing factors.

In the present study, the composition of lignans (sesamin, sesamolin, and sesamol) was also determined, and the results showed that the levels of sesamin and sesamolin were significantly lower in black sesame seeds than in white sesame seeds, despite black sesame exhibiting the highest TPC and antioxidant capacity. In white sesame seeds, sesamin and sesamolin concentrations were 353.5 mg/100 g seed DW and 154.3 mg/100 g seed DW, respectively. In comparison, black sesame seeds contained only 120.5 mg/100 g seed DW of sesamin and 113.9 mg/100 g of sesamolin. Sesamol compound was not detected in the fresh white and black sesame seed samples analyzed. This indicates that sesamol was either absent or present at concentrations below the detection limit of the analytical method used in this study.

Overall, white sesame seeds demonstrated superiority in oil yield, sesamin, and sesamolin content, while black sesame excelled in TPC and antioxidant capacity.

#### 2.3.2. Roasted White and Black Sesame Seeds

White and black sesame seeds roasted at 200 °C for 15 min were analyzed for key phytoconstituents (TPC, antioxidant activity, and lignans) and compared with fresh seeds (Table 4). The roasted black sesame retained higher TPC (105 mg GAE/100 g DW), FRAP (185.5 mg TE/100 g DW), and CUPRAC (859.0 mg TE/100 g DW) than roasted white sesame, which recorded values of 79.3 mg GAE/100 g DW, 167.0 mg TE/100 g DW, and 442.7 mg/100 g DW, respectively. These values reinforce the trend already observed in fresh seeds, where black sesame showed superior TPC and antioxidant potential.

While comparing the TPC and antioxidant capacity of roasted and unroasted sesame seeds, roasting altered these values in a distinct way (Table 4). The value of TPC decreased slightly in roasted sesame seeds, while the FRAP and CUPRAC values increased. In white sesame, roasting reduced TPC by about 25%, and increased FRAP and CUPRAC by roughly 20% and 3%, respectively. In black sesame, TPC decreased by about 17%, while FRAP and CUPRAC rose by approximately 16% and 17%, respectively.

Few previous results align with those obtained in the present study [38,39]. For example, Akele et al. [38] investigated TPC and antioxidant capacity in unroasted and roasted sesame seeds from the different sources in Ethiopia and observed reduced TPC but significantly increased antioxidant activity. However, other studies have reported results differing from those obtained in the present study. For example, Chen et al. [40] investigated six varieties of unroasted and roasted sesame seeds (roasted at 240 °C for 20 min) and recorded lower TPC in unroasted samples (121.7–432.4 mg GAE/100 g DW) compared with roasted ones (786.13–1089.54 mg GAE/100 g DW). Antioxidant activities measured by DPPH (2,2-diphenyl-1-picrylhydrazyl), ABTS (2,2′-azino-bis (3-ethylbenzthiazoline) 6-sulphonic acid), FRAP, and ACI (antioxidant potency composite index) assays also increased markedly on roasted seeds, with improvements ranging from 53.6–211.8%, 1.3–383.9%, 44.1–137.7%, and 29.8–216.6%, respectively [40]. Salamatullah et al. [41] also evaluated TPC and antioxidant capacity in both unroasted and roasted sesame seeds. Roasting was carried out using a microwave oven at 360, 540, and 720 W for 5, 10, and 15 min, as well as an electric oven at 180 °C, 200 °C, and 220 °C for 10, 20, and 30 min. Their findings showed that all roasted samples exhibited higher TPC and antioxidant capacity compared to unroasted seeds [41]. Few other studies have also reported increased TPC and antioxidant capacity in roasting up to a certain temperature and duration compared with unroasted seeds, although the optimal conditions vary across studies. For example, El Hanafi et al. [31] observed better performance in antioxidant capacity and TPC of sesame seeds while roasting them at 140 °C/40 min; however, these values decreased at subsequent roasting temperatures. Jannat et al. [42] also roasted sesame seeds at 180 °C, 200 °C, and 220 °C for 10–20 min and found higher TPC and antioxidant capacity (FRAP) in all roasted samples compared to unroasted ones. However, values declined beyond 200 °C/20 min. Such inconsistent results highlight the need for further studies on individual phenolics and other phytoconstituents profiling of unroasted and roasted seeds and identifying the specific compounds responsible for antioxidant activity during roasting.

In the present study, the roasted white sesame seeds contained 294.0 mg/100 g DW of sesamin and 133.6 mg/100 g DW of sesamolin, whereas roasted black sesame seeds contained 117.9 mg/100 g DW of sesamin and 104.7 mg/100 g DW of sesamolin (Table 4). These values further support the trend observed in fresh seeds, with white sesame exhibiting higher sesamin and sesamolin.

While comparing the sesamin and sesamolin values of unroasted and roasted sesame seeds, it was noted that roasting also influenced lignan content (Table 4). In white sesame, sesamin decreased by 59.5 mg/100 g DW and sesamolin by 20.7 mg/100 g DW, which corresponds to losses of about 17% and 13%. In black sesame, sesamin and sesamolin decreased by 2.6 mg/100 g DW and 9.2 mg/100 g DW, respectively, which corresponds to losses of roughly 2% and 8%. These results suggest that although there was slight degradation of sesamin and sesamolin in roasted seeds, the loss of these lignans were higher in white sesame seeds than in black ones. Similar to the fresh samples, sesamol was not detected in any roasted sesame seed samples.

Few studies have been done in the past to understand the lignans in unroasted and roasted sesame seeds, and their reports showed the decrease of sesamin and sesamolin compounds and an increase of sesamol in roasted seeds. For example, Chen et al. [40] investigated six varieties of unroasted and roasted sesame seeds (roasting at 240 °C for 20 min) and observed a reduction in sesamin and sesamolin content and increase of sesamol in the roasted sesame seeds compared to the unroasted ones. Comini et al. [43] examined sesamin, sesamolin, and sesamol in unroasted and roasted (180 °C and 250 °C for 25 min) white and black sesame seeds. They found a slight decrease in sesamin and sesamolin at 180 °C for both varieties, while at 250 °C, these compounds declined significantly. At this higher temperature, sesamolin was no longer detected, having been converted into sesamol, which showed an increased concentration [40].

Overall, this study showed that roasting at 200 °C for 15 min affected the phytoconstituents of sesame seeds, leading to reduced TPC, sesamin, and sesamolin levels, but to higher antioxidant capacity. This finding suggests that lignans (sesamin and sesamolin) have week antioxidant capacity. To further confirm the relationship of sesamin, sesamolin, and sesamol with TPC and antioxidant capacity (CUPRAC and FRAP), different concentrations of pure sesamin, sesamolin, and sesamol compounds were prepared and tested (Table 5 and Table 6). As seen in Table 5, there was little increment in the values of TPC and antioxidant capacity with the increase of sesamin and sesamolin concentrations from 50 mg/L to 200 mg/L. For instance, the TPC values for sesamin increased marginally from 9.0 to 12.2 mg GAE/L, while those for sesamolin increased from 9.3 to 16.0 mg GAE/L. Similarly, FRAP values remained very low, increasing only from 0.3 to 1.6 mg TE/L for sesamin and from 1.2 to 3.6 mg TE/L for sesamolin. CUPRAC values also showed only modest increases with concentration. These results indicate that sesamin and sesamolin possess relatively weak reducing power and limited contribution to antioxidant capacity despite increasing concentration. Hoyos, et al. [44] also observed no significant correlation of sesamin and sesamolin with CUPRAC and FRAP; however, they observed a moderate positive correlation with TPC while investigating different varieties of sesame in Queensland, Australia.

In contrast, sesamol demonstrated a much stronger antioxidant response (Table 6). As the concentration of sesamol increased from 10 to 70 mg/L, TPC values increased markedly from 17.3 to 66.9 mg GAE/L. Similarly, FRAP values increased sharply from 33.7 to 226.5 mg TE/L, and CUPRAC values increased from 44.0 to 238.3 mg TE/L. The rapid increase in antioxidant capacity with increasing sesamol concentration indicates that sesamol has a significantly higher antioxidant potential than sesamin and sesamolin.

### 2.4. Phytochemistry in Fresh and Roasted White and Black Sesame Seed Oils Screw-Pressed at Different Temperatures

#### 2.4.1. Fresh White and Black Sesame Oil

The TPC and antioxidant capacity of oils extracted from fresh white and black sesame seeds at different screw-press temperatures were assessed (Table 7). The TPC and antioxidant capacity were consistently higher in black sesame oil than in white sesame oil at all temperatures. In black sesame oil, the TPC, CUPRAC, and FRAP values ranged from 54.6–28.5 mg GAE/100 g DW, 28.4–18.9 mg TE/100 g DW, and 217.6–137.7 mg TE/100 g DW, respectively. In comparison, white sesame oil showed lower values of 44.7–24.5 mg GAE/100 g DW, 25.1–17.9 mg TE/100 g DW, and 90.8–65.1 mg TE/100 g DW, respectively. The higher TPC and antioxidant capacity observed in black sesame seeds in this study suggest that seeds naturally richer in phenolics and antioxidants tend to yield oils with proportionally higher concentrations of these compounds.

Table 7 also reveals a steady decline in TPC and antioxidant capacity of the oils with increasing screw-press temperature. From 40 °C to 200 °C, the TPC of white sesame oil decreased by 20.2 mg GAE/100 g DW, representing about a 45% decrease. Black sesame oil showed an even larger drop of 26.1 mg GAE/100 g DW, roughly 48%. Similarly, FRAP values also fell over the same temperature range, decreasing by 7.2 mg TE/100 g DW in white sesame oil (about 29%) and by 9.5 mg TE/100 g DW in black sesame oil (about 33%). The reduction was more noticeable in the CUPRAC assay. White sesame oil declined by 25.7 mg TE/100 g DW (about 28%), while black sesame oil dropped by 79.9 mg TE/100 g DW (about 37%). These results indicate that higher screw-press temperatures may cause thermal degradation of phenolic compounds and antioxidants, thereby reducing the overall antioxidant capacity of the sesame oils. Similar results have been reported by some researchers, including Piravi-Vanak et al. [10]. For example, Piravi-Vanak et al. [10] investigated the TPC in the oils of two sesame varieties (Darav and Tak Shakh Naz) extracted at four screw-pressing temperatures (30, 60, 90, and 120 °C). They reported a decrease in TPC with increasing temperature, from 395.6 to 335.5 mg/kg in Darav and from 413.0 to 364.0 mg/kg in Tak Shakh Naz [10].

The concentrations of sesamin, sesamolin, and sesamol in the oil were analyzed (Table 8). As shown, white sesame oil contained higher levels of sesamin and sesamolin compounds than black sesame oil across all screw-press temperatures. In white sesame oil, sesamin ranged from 456.7 mg/100 g DW at 40 °C to 446.3 mg/100 g DW at 200 °C, a reduction of about 2.3%. In black sesame oil, sesamin declined from 182.2 mg/100 g DW to 173.2 mg/100 g DW, which is about a 4.9% decrease. Sesamolin also followed a similar pattern. In white sesame oil, it dropped from 218.8 mg/100 g DW at 40 °C to 212.9 mg/100 g DW at 200 °C, a decrease of roughly 2.7%. In black sesame oil, sesamolin declined from 188.6 mg/100 g DW to 181.3 mg/100 g DW, about a 3.9% reduction.

Although sesamol was not detected in fresh white and black sesame seed extracts in the present study, it was detected in the corresponding oil samples. This discrepancy in sesamol detection between seed and oil matrices may be attributed to methodological differences in the extraction procedures, particularly variations in sample-to-solvent ratio and matrix composition, which can influence the efficiency of compound recovery and, consequently, the detectability of sesamol.

When comparing the three investigated lignans, the contribution of sesamol in fresh white and black sesame oil was extremely low; however, it exhibited a distinct pattern. Sesamol levels were slightly higher in black sesame oil than in white sesame oil across all screw-press temperatures, with a gradual increase observed as processing temperature increased. In white sesame oil, sesamol content rose from 7.5 mg/100 g DW at 40 °C to 9.3 mg/100 g DW at 200 °C, representing an increase of approximately 24.0%. In black sesame oil, it increased from 9.5 mg/100 g DW to 10.3 mg/100 g DW, corresponding to a smaller rise of about 8.4%.

Overall, although the concentrations of sesamin and sesamolin slightly decreased and sesamol slightly increased with increasing screw-press temperature in both white and black sesame oils, the changes were not statistically significant. This suggests that these lignans are relatively stable under the range of processing temperatures used, and that thermal effects may have only a minor influence on their retention during screw-press extraction.

The composition of fatty acids, saturated fatty acids (SFAs), monounsaturated fatty acids (MUFAs), and polyunsaturated fatty acids (PUFAs), in the sesame oils obtained from screw-pressed unroasted seeds was assessed at different temperatures (40–200 °C) (Table 9). As shown, a total of 6 fatty acids, two each from SFAs (palmitic acid and stearic acids), MUFAs (oleic acid, eicosenoic acid), and PUFAs (linoleic acid and α-linolenic acid) were detected, where oleic and linoleic acids were predominant components. The fatty acid composition of white and black sesame oils extracted at all the screw-press temperatures from 40–200 °C remained relatively stable, although minor fluctuations were observed, indicating that temperature had a limited impact on the overall fatty acid profile. These findings align with previous reports, including Piravi-Vanak et al. [10].

Both oils were dominated by unsaturated fatty acids (UFAs), particularly oleic (C18:1) and linoleic (C18:2) acids, which together accounted for 84.0–84.9% in white sesame oil and 83.7–84.6 in black sesame oils of total fatty acids across all extraction temperatures. The MUFAs and PUFAs both showed approximately similar contributions in total UFAs. MUFAs in white and black sesame were in the range between 42.7–43.3% and 42.9–43.4%, respectively, while PUFAs were 40.7–42.1% and 40.8–41.2%, respectively. Among the SFAs, palmitic and stearic acids were the major constituents. The total content of SFAs ranged from 14.2–15.5% in white sesame oil and 14.8–16.1% in black sesame oil.

Overall, the combined proportion of MUFAs and PUFAs (≈83–85%) in white and black sesame oils was substantially higher than the SFA fraction, resulting in a high UFA/SFA ratio (5.2–6.0). This ratio reflects the favorable nutritional quality and oxidative stability of sesame oil, consistent with previous reports highlighting its health-promoting lipid profile. The stable fatty acid composition across the screw-pressed temperature range further suggests that moderate thermal processing during mechanical extraction does not substantially degrade the essential fatty acids in sesame oil.

#### 2.4.2. Roasted White and Black Sesame Oil

The TPC and antioxidant capacity of oils extracted from roasted black and white sesame seeds were assessed at 40 °C to 200 °C screw-press temperatures (Table 10). Across all parameters, a declining trend was observed as the screw-press temperature increased. The TPC dropped by 15.8 mg GAE/100 g oil DW, which is about a 45.5% decrease in roasted white sesame oil, while black sesame oil showed a reduction of 18.8 mg GAE/100 g oil DW, equal to roughly 43.1%. For FRAP, the decrease was 11.5 mg TE/100 g oil DW, accounting for about a 32.2% loss in roasted white sesame oil, while the reduction in roasted black sesame oil was 14.1 mg TE/100 g oil DW, equal to roughly 35.8% loss. For CUPRAC, reduction was 28.2 mg TE/100 g oil DW or about 28.4% in roasted white sesame oil, and for roasted black sesame oil, it was 63.3 mg TE/100 g oil DW, which is about 26.1%. This pattern of loss is consistent with the results obtained from unroasted seed oils, suggesting that increasing screw-press thermal intensity applied to the seeds in the present study contributes to phenolic degradation regardless of roasting.

While comparing the TPC and antioxidant capacity of unroasted and roasted sesame seed oil, screw-pressed at different temperatures, the TPC decreased in roasted sesame seed oil, whereas FRAP and CUPRAC increased (Table 7 and Table 10). For example, the TPC, FRAP, and CUPRAC in roasted white sesame seed oil, screw-pressed at 40 °C, were 34.7 mg GAE/100 g oil DW, 32.7 mg TE/100 g oil DW, and 99.3 mg TE/100 g oil DW, respectively, while these values for unroasted white sesame seed oil, screw-pressed at 40 °C, were 44.7 mg GAE/100g oil DW, 25.1mg TE/100 g oil DW, and 90.8 mg TE/100 g oil DW, respectively. A similar trend was also seen in the seeds themselves. Inconsistent results have been observed in previous studies. For example, El-Beltagi et al. [45] investigated oil samples extracted from both unroasted and roasted sesame seeds, where roasting was carried out using various methods, including a cooker oven, stovetop pan, microwave, and electric frying pan. They found that roasted seeds exhibited higher TPC and stronger antioxidant activity compared to unroasted seeds. Furthermore, Arab et al. [30] investigated oils obtained from unroasted and roasted sesame seeds (roasted at 150, 180, 210, 250, 300 °C), and they also observed higher TPC and antioxidant activity in roasted sesame oil than in unroasted sesame oil. The antioxidant activity increased significantly throughout each roasting temperature; however, TPC increased only until 180 °C roasting temperature and thereafter started decreasing [30]. Hamitri-Guerfi et al. [32] investigated the oils obtained from unroasted and roasted sesame seeds at 180 °C for 20 min and observed higher levels of TPC and antioxidant activity in the oil of roasted seeds than in unroasted seeds.

The lignans, sesamin, sesamolin, and sesamol, of oils extracted from roasted black and white sesame seeds at 40 °C to 200 °C screw-press temperatures were also investigated (Table 11). As shown in the table, a slight decrease in sesamin and sesamolin with the increase of screw-press temperatures in roasted seed oils was observed, whereas sesamol exhibited a slight increasing trend. A similar trend was also seen in the unroasted seed oils.

Specifically, in roasted white sesame oil, sesamin decreased from 438.4 to 429.3 mg/100 g oil DW and sesamolin from 208.9 to 199.7 mg/100 g oil DW as the screw-press temperature increased from 40 °C to 200 °C. In roasted black sesame oil, sesamin decreased from 166.8 to 151.0 mg/100 g oil DW, and sesamolin from 175.7 to 168.7 mg/100 g oil DW over the same temperature range. In contrast, the sesamol content increased slightly with increasing temperature, rising from 22.1 to 25.8 mg/100 g oil DW in white sesame oil and from 25.2 to 27.7 mg/100 g oil DW in black sesame oil. However, these variations were not statistically significant (*p* > 0.05), indicating that the lignans remained relatively stable across the tested processing temperatures.

Lee et al. [46] also reported little degradation of sesamin and sesamolin while heating the oil sample at 180 °C for 1 h in an oven, showing that the sesamin and sesamolin are relatively heat-stable. Arab et al. [30] investigated the oils of unroasted and roasted (120, 150, 180, 210, 250, and 300 °C for 20 min) white sesame seed and reported decreasing sesamin from 393.25 mg/100 g to 357.80 mg/100 g and sesamolin from 202.92 mg/100 g to 147 mg/100 g at 300 °C. Furthermore, Ji et al. [47] roasted sesame seeds at three different temperatures (160 °C, 180 °C, and 200 °C) for the duration of 20, 40, and 60 min to compare sesamin, sersamolin, and sesamol in roasted and unroasted seeds, and observed that roasting did not affect sesamin, but it caused sesamolin to decrease and sesamol to increase significantly in sesame oil.

The fatty acids of oils extracted from roasted black and white sesame seeds at 40 °C to 200 °C screw-press temperatures (Table 12). As shown, there was no notable change in fatty acid composition for both temperatures. Furthermore, the study also observed no significant change in fatty acid composition while comparing the oils obtained from roasted and unroasted black and white sesame seeds (Table 9 and Table 12).

The fatty acid studies have been done on unroasted and roasted sesame seeds to understand the effect of roasting temperature on fatty acid composition, and diverse results have been reported in the literature. On the one hand, authors have reported a significant difference in fatty acids between the roasted and unroasted sesame oils [30,41,48]. Comini et al. [43] examined fatty acids in unroasted and roasted (180 °C and 250 °C) white and black sesame seeds. They observed that the roasting process modulates the fatty acid profile by increasing the content in UFAs, in the trans configuration mainly.

On the other hand, several studies have documented minimal or no change in fatty acid composition following roasting. Ji et al. [47] roasted sesame seeds at three different temperatures (160 °C, 180 °C, and 200 °C) for the duration of 20, 40, and 60 min) to compare fatty acids in roasted and unroasted seeds. They observed no obvious differences in the fatty acid profile of both unroasted and roasted seed oils. Hamitri-Guerfi et al. [32] also didn’t observe clear differences in the fatty acid profile between roasted (roasted at 180 °C for 20 min) and unroasted sesame oils. This suggests further studies are needed to understand how factors such as variety, moisture content, roasting method, and oxidation influence lipid stability during roasting.

## 3. Materials and Methods

### 3.1. Chemicals and Reagents

All reagent-grade chemicals applied in this study were purchased from ChemSupply (Gillman, SA, Australia) or Sigma-Aldrich (Melbourne, VIC, Australia). The Milli-Q^®^ water (Hach Pacific, QLD, Australia) was applied while needed in solution preparation or chemical analysis. Chemical preparations or chemical reagents were stored at 4 °C in the dark before application.

### 3.2. Sample Collection

Two different varieties of sesame seeds, white (variety: WHITE 3) and black (variety: BLACK 5), were chosen for the present study. The white and black sesame seeds for the study were provided by AgriVentis Technologies Pty Ltd. (https://www.agriventistechnologies.com.au (1 Macquarie Place, Sydney, NSW, Australia)). White sesame was sown on 20 January 2025 in Emerald, QLD, Australia (23°33′55.9″ S; 148°13′52.3″ E). These plants were grown under irrigated conditions and, after maturing, were harvested on 20 May 2025. The black sesame crop was sown on 14th September 2024 in Tully (18°06′53.0″ S 145°54′44.5″ E), QLD, Australia. These plants were grown under dryland conditions, and after ripening, were harvested on 3 January 2025. Both white and black sesame seeds harvested from these trials were cleaned with a seed cleaner (MK3-Kimseed, Wangara, AustraliaMK3; Kimseed, Perth, WA, Australia), color sorted three times with a color sorter (Topsort, TS16, Hefei, Anhui, China), zip-locked and stored in a cold room at 4 °C until further analysis.

### 3.3. Roasting of Seeds

Roasting temperature, time, and method strongly affect sesame oil yield and quality, and studies show that moderate roasting between 160 and 210 °C for about 10–25 min gives the best balance of flavor, nutrition, and oil recovery [30,41,49]. Based on this evidence, the seeds in this study were first spread homogenously into the aluminum plate and then roasted at 200 °C for 15 min using an air dryer (Cole-Parmer^®^ OVG-400 Series Gravity Convection Drying Oven, Vernon Hills, IL, USA) for further analysis:

### 3.4. Moisture Content Analysis

The cleaned white and black sesame seed samples were freeze-dried using the protocol applied earlier by Johnson et al. [50]. Briefly, all samples were stored at −80 °C for 48 h prior to freeze-drying and subsequently put in a freeze dryer (FTS System-Flexi dry MP freeze drier, New York, NY, USA), maintaining the temperature and pressure between −45 °C to −50 °C and 60 to 100 mTorr, respectively, for 7 days. The moisture content was finally determined using the following formula:


$$Moisture\;content\;\left(\%\right)=\frac{fresh\;weight\;of\;sample-freeze\;dried\;weight\;of\;sample)}{fresh\;weight\;of\;sample}\times 100$$

### 3.5. Soxhlet Extraction

Soxhlet extraction with *n*-hexane is widely regarded as the standard method for determining the total extractable oil (total lipid content) in seeds, as it provides the true or near-complete oil content [51,52,53,54]. Therefore, the method applied by Saxena et al. [54] was adopted with slight modification to measure the total oil yield of sesame seeds in the present study. Briefly, 5 g of finely ground sesame seeds were weighed and placed into a thimble (Ankom bag, ANKOM technology, Macedon, NY, USA) and sealed. The thimble was then put in the Soxhlet apparatus and extracted with 150 mL of *n*-hexane for 3 days (72 h). The oil extract was concentrated using rotavapor, and the solvent-free oil was measured to evaluate the total extractable oil content using the formula given below, and results were expressed on DW basis.


$$Total\;extractable\;oil\;content\;\left(\%\right)=\frac{Final\;weight\;of\;flask-Empty\;weight\;of\;flask}{Dry\;weight\;of\;seed\;sample\;used\;for\;extraction}\times 100$$

### 3.6. Screw-Press Oil Extraction

**Figure 1:**
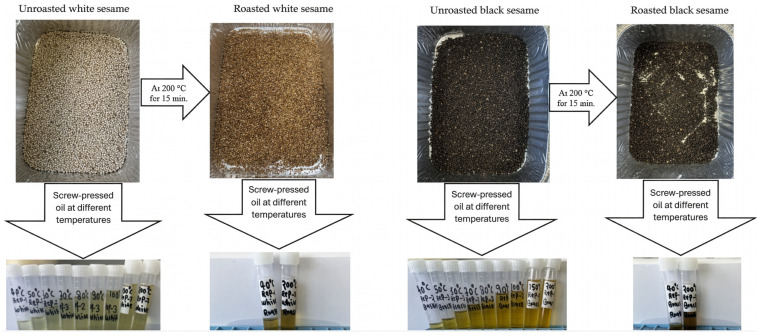
Extraction of oils from roasted and unroasted white and black sesame seeds at different screw-press temperatures.

The fresh or unroasted sesame seeds were screw-pressed at nine various temperatures (40, 50, 60, 70, 80, 90, 100, 150, and 200 °C) while keeping the moisture content and feeding rate constant (Figure 1). Sesame seeds were screw-pressed to obtain oil, following the protocol of Thani et al. [55]. Sesame seeds (20 g) were pressed in a temperature-controlled automatic oil press machine (Wgwioo brand; 110 V/220 V, 600–1500 W, 42 × 16 × 32 cm^3^) with a fixed shaft speed of 58 rpm and a feeding rate of 20 g/min. Previous studies have indicated that moisture content in the seed is crucial for optimal oil recovery [36,56]. Olayanju [57] reported that a moisture level of 5.3% with a worm-shaft speed of 45 rpm produced the best oil and cake quality. Therefore, the seed moisture content was maintained at approximately 5.3%. For this, the samples were first stored at −80 °C for 48 h and subsequently freeze-dried using a freeze-drier (FTS system-Flexi dry MP freeze-drier, New York, NY, USA), maintaining temperature and vacuum pressure approximately −50 °C and 100 mTorr, respectively, for 7 days to remove all the moisture from the seeds. The seeds were then adjusted with Milli-Q^®^ water to reach 5.3% moisture (using a mass balance equation given below), sealed in plastic bags, mixed thoroughly, and stored at 4 °C for 24 h. The conditioned seeds were then screw-pressed at the desired temperature by feeding the sample into the hooper at a constant rate (20 g/min). Although the feeding rate was maintained at 20 g/min, the material underwent continuous compression and sufficient residence time within the screw chamber, ensuring adequate exposure to the set processing temperature. To maximize oil recovery, the motor was briefly operated in reverse after completion of the forward pressing cycle to expel any residual material and extract remaining oil. Roasted sesame seeds were processed using the same procedure mentioned above; however, only two screw-press temperatures (40 °C and 200 °C) were applied. In this case, moisture balance calculations were not performed. The extracted oil coming from unroasted and roasted seeds was collected in individual 10 mL centrifuge tubes and centrifuged at 3000× *g* for 20 min. Following centrifugation, the sediment formed a sludge at the bottom of the tubes, while the clarified oil remained above. The oil phase was carefully transferred into fresh 10 mL centrifuge tubes. Residual oil adhering to the original tubes was recovered by rinsing with *n*-hexane, leaving only the sediment behind. Oil yield was subsequently calculated using a given formula, and results were expressed on a DW basis.


$$Mass\;balance\;equation=\frac{\left(target\;moisture\;\%-initial\;moisture\;\%\right)}{100}\times \left(seed\;weight\right)$$

$$Oil\;yield\;(\%)=\frac{weight\;of\;oil}{dry\;weight\;of\;seed\;used\;for\;screw\;press\;extraction}\times 100$$

### 3.7. Extraction of Seeds and Oil Samples to Determine TPC, Antioxidant Capacity, and Lignans

Sesame seed extracts were prepared using an aqueous-methanolic extraction method previously developed in our laboratory [16,44,55,58]. Briefly, freeze-dried seed powder (0.5 g) was mixed with 7 mL of 90% methanol in a centrifuge tube and vortexed for 10 s, followed by extraction using an end-over-end shaker (Ratek RM4, Melbourne, VIC, Australia) for 60 min at 50 rpm. The extracted sample was then centrifuged for 10 min at 1000× *g* (Heraeus X1 Multifuge, Thermo Fisher Scientific, Melbourne, VIC, Australia), and the supernatant was collected. The extraction was repeated on the residue with another 7 mL of solvent for 20 min in an end-over-end shaker, and the supernatants were combined, adjusted to 14 mL, and stored in a refrigerator at 4 °C until analysis.

Oil extraction followed the same procedure applied in our previous study [16]. Briefly, screw-pressed oil (1 g) was mixed with 1 mL of hexane and then with 7 mL of 90% methanol, vortexed for 10 s, extracted with an end-over-end shaker for 1 h, and centrifuged; the methanolic supernatant was collected. The residue was re-extracted with another 7 mL of 90% methanol, and the combined extracts were adjusted to 14 mL and stored at 4 °C until phytoconstituent analysis.

### 3.8. Experimental Analysis

#### 3.8.1. TPC

The Folin–Ciocalteu (FC) method was used to determine the TPC of the samples following the same steps applied in our previous study [55]. Briefly, 400 µL of extract was reacted with FC reagent and sodium carbonate, incubated at 40 °C for 30 min, and absorbance was measured at 760 nm using a spectrophotometer (Thermo Scientific Genesys 10S UV–Vis Spectrophotometer, Madison, WI, USA). TPC was calculated from a gallic acid standard curve and expressed as mg GAE/100 g DW.

#### 3.8.2. Antioxidant Capacity

##### FRAP Analysis

The FRAP analysis was conducted following the same procedures applied in our previous study [55]. Briefly, fresh reagent (acetate buffer, TPTZ (2,4,6-tris(2-pyridyl)-s-triazine), and ferric chloride, 10:1:1) was prepared. A 100 µL of sample was mixed with 3 mL reagent, incubated at 37 °C for 4 min, and absorbance was measured at 593 nm using a spectrophotometer (Thermo Scientific Genesys 10S UV–Vis Spectrophotometer, Madison, WI, USA), with results expressed as mg TE/100 g DW based on a Trolox standard curve.

##### CUPRAC Analysis

The analysis of CUPRAC was conducted following the same procedures applied in our previous study [55]. Briefly, copper (II) chloride, ammonium acetate, and neocuproine solutions were mixed with water, and the sample extract was incubated at 50 °C for 30 min, and absorbance was measured at 450 nm using a spectrophotometer (Thermo Scientific Genesys 10S UV–Vis Spectrophotometer, Madison, WI, USA). The results were calculated from a Trolox standard curve and expressed as mg TE/100 g DW.

#### 3.8.3. Lignans Analysis

**Figure 2:**
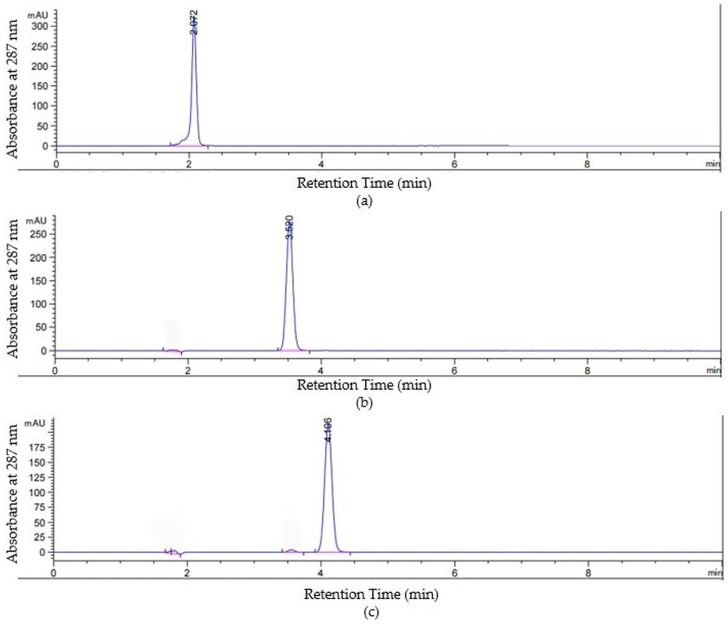
(**a**) A chromatogram of sesamol standard (100 mg/L), (**b**) a chromatogram of sesamin standard (100 mg/L), and (**c**) a chromatogram of sesamolin standard (100 mg/L).

The lignans (sesamin, sesamolin, and sesamol) in seed and oil extracts were quantified by high-performance liquid chromatography following the procedures applied by Hoyos et al. [44]. Briefly, an Agilent 1100 system (G1313A autosampler, G1322A vacuum degasser, G1311A quaternary pump, and G1365B multi-wavelength detector module; Agilent Technologies, Santa Clara, CA, USA) was used with an Agilent Eclipse XDB-C18 column (dimensions: 150 × 4.6 mm; 5 µm particle size; Agilent Technologies, Santa Clara, CA, USA). The injection volume was 10 µL, and an isocratic mobile phase of water: methanol (20:80) was fixed at 0.8 mL/min, while the total run time was 10 min. Fixing ultraviolet detection at 287 nm, compounds were identified by the retention times of standards and quantified using external calibration curves (0–300 mg/L), with results expressed as mg/100 g DW. The high-performance liquid chromatograms of sesamol (a), sesamin (b), and sesamolin (c) standards (100 mg/L) are presented in Figure 2 as representative examples. In each chromatogram, the predominant peak (highlighted in blue) corresponds to the respective target compound.

The stock and standard solutions of pure sesamin, sesamolin, and sesamol compounds were prepared with methanol in the present study, and the different concentrations of these standards were also tested against TPC, FRAP, and CUPRAC to understand the correlation among them.

#### 3.8.4. Fatty Acids Analysis

Fatty acid methyl esters (FAMEs) were prepared following the protocol reported by O‘Fallon et al. [59] and modified by Hoyos et al. [44] and Thani et al. [55]. Briefly, seed oil (0.02 g) was mixed with reagents, sodium hydroxide prepared with methanol, and an aqueous sodium bicarbonate, and then extracted with hexane, washed, filtered, and diluted before analysis. The FAMEs were then analyzed by Gas Chromatography–Mass Spectrometry (Shimadzu QP2010 Plus, Kyoto, Japan) using a Restek FAMEWAX capillary column (Restek Corporation, Bellefonte, PA, USA) (30 m × 0.32 mm I.D. × 0.25 µm thickness). Injection volume was 0.5 µL in split mode, where the split ratio was 10. Helium was used as a carrier gas with a flow rate of 2 mL/min, and the total run time was 35 min. The identification is based on mass spectra and comparison with FAME standards. The results of individual fatty acids were expressed as a percentage of the total fatty acids present in the sample.

### 3.9. Statistical Analysis

The Soxhlet extraction and the analysis of phytoconstituents in seeds were carried out with two replicates, while the screw-press oil extraction and the analysis of phytoconstituents in screw-pressed oil were carried out with three replicates. The results were illustrated as mean ± standard deviation (SD). Data were analyzed using one-way ANOVA in IBM SPSS version 28.000. Differences were considered statistically significant at *p* < 0.05. In the tables presented in Section 2, values within the same column followed by the same superscript letter indicate that the values are not significantly different (*p* > 0.05).

## 4. Conclusions

This study evaluated the total extractable oil (total lipid content) and important health-benefitting phytoconstituents in white and black sesame seeds, as well as the influence of screw-press temperatures on oil yield and health-benefiting phytoconstituents of unroasted and roasted black and white sesame oil, with the aim of identifying optimal processing conditions for qualitative and quantitative oil production.

Higher amounts of TPC, antioxidant activity, and sesamol were found in black sesame, but the white ones were superior when it came to sesamin, sesamolin, total lipid content, and screw-press oil yield.

In unroasted black and white sesame seeds, the oil extraction increased with an increase in screw-press temperatures up to 150 °C, but a slight decrease or no further increase occurred when it reached 200 °C, indicating thermal limitations at excessive temperatures. While comparing the screw-pressed oil yield of roasted and unroasted sesame seeds, the roasting process resulted in a decrease in the efficiency of oil extracted by mechanical presses, likely due to excessive moisture loss.

While comparing TPC, antioxidant activity, sesamin, sesamolin, sesamol, and fatty acid composition on unroasted and roasted sesame seeds, as well as derived oils coming from different screw-press temperatures, different levels of effects on phytoconstituents were observed due to these temperatures; however, sesamin, sesamolin, sesamol, and fatty acid composition were comparatively stable with the applied screw-press temperatures.

Overall, these findings support the use of moderate screw-press temperatures to provide the best balance between maximizing oil yield and preserving bioactive compounds.

## Figures and Tables

**Table 1:** Total extractable oil and solid residue yield in Soxhlet extracted fresh white and black sesame seeds.

Seed Samples	Total Extractable Oil Yield %
White sesame seeds	50.9 ± 0.4
Black sesame seeds	48.1 ± 0.7
*p*-value	*

Results are presented as average values ± SD (*n* = 2). The symbol “*” indicates statistical significance at *p* < 0.05.

**Table 2:** Oil, sludge, and seedcake yield in fresh white and black sesame seeds under different levels of screw-press temperature.

Screw-Press Temperature	Oil Yield %		Sludge Yield %		Seedcake %	
	White Seed	Black Seed	White Seed	Black Seed	White Seed	Black Seed
40 °C	18.5 ± 1.7 ^a^	11.6 ± 0.8 ^a^	10.8 ± 1.1 ^e^	11.7 ± 0.9 ^d^	70.7 ± 1.5 ^d^	76.8 ± 0.2 ^c^
50 °C	21.9 ± 1.9 ^ab^	14.1 ± 1.4 ^a^	9.1 ± 0.7 ^d^	10.7 ± 1.0 ^cd^	68.9 ± 2.4 ^cd^	75.2 ± 2.2 ^c^
60 °C	23.8 ± 2.0 ^ab^	20.3 ± 1.5 ^b^	9.0 ± 0.1 ^d^	10.0 ± 0.5 ^cd^	67.1 ± 1.9 ^bcd^	69.7 ± 1.6 ^b^
70 °C	26.5 ± 2.4 ^bc^	23.3 ± 2.2 ^bc^	8.8 ± 0.7 ^d^	9.4 ± 0.6 ^c^	64.7 ± 1.8 ^abc^	67.4 ± 2.4 ^ab^
80 °C	29.7 ± 2.6 ^cd^	25.1 ± 1.9 ^bcd^	6.9 ± 0.6 ^c^	7.4 ± 0.7 ^b^	63.5 ± 3.2 ^abc^	67.5 ± 1.4 ^ab^
90 °C	32.0 ± 2.3 ^cde^	27.1 ± 1.2 ^cde^	4.9 ± 0.4 ^b^	6.9 ± 0.5 ^b^	63.1 ± 2.1 ^ab^	66.1 ± 0.8 ^ab^
100 °C	34.3 ± 2.3 ^de^	29.1 ± 2.7 ^def^	4.4 ± 0.4 ^ab^	6.2 ± 0.6 ^ab^	61.3 ± 2.0 ^a^	64.7 ± 2.2 ^a^
150 °C	36.8 ± 1.3 ^e^	32.6 ± 2.5 ^f^	3.3 ± 0.2 ^a^	4.8 ± 0.4 ^a^	59.9 ± 1.1 ^a^	62.6 ± 2.1 ^a^
200 °C	36.6 ± 1.1 ^e^	31.1 ± 1.8 ^ef^	3.1 ± 0.2 ^a^	4.7 ± 0.3 ^a^	60.3 ± 1.1 ^a^	64.2 ± 1.6 ^a^

Results are presented as average values ± SD (*n* = 3). Values followed by identical superscript letters along the column are statistically similar.

**Table 3:** Oil, sludge, and seedcake yields of roasted white and black sesame seeds while screw-pressing under different levels of temperatures.

Screw-Press Temperature of Roasted Seeds	Oil Yield %		Sludge Yield %		Seedcake %	
	White Seed	Black Seed	White Seed	Black Seed	White Seed	Black Seed
40 °C	12.7 ± 2.3	7.7 ± 1.7	12.6 ± 1.2	16.3 ± 2.5	74.7 ± 3.1	75.9 ± 2.0
200 °C	32.5 ± 2.5	27.9 ± 5.4	7.8 ± 2.2	9.0 ± 2.4	59.7 ± 4.0	63.1 ± 6.6
*p*-value	*	*	*	*	*	*

Results are presented as average values ± SD (*n* = 3). The symbol “*” indicates statistical significance at *p* < 0.05.

**Table 4:** Chemical characteristics in fresh and roasted white and black sesame seeds.

Seed Samples	TPC (mg GAE/100 g Seed DW)	FRAP (mg TE/100 g Seed DW)	CUPRAC (mg TE/100 g Seed DW)	Sesamin (mg/100 g Seed DW)	Sesamolin (mg/100 g Seed DW)	Sesamol (mg/100 g Seed DW)
Fresh white sesame	106.6 ± 4.1 ^b^	138.9 ± 3.0 ^a^	428.3 ± 11.6 ^a^	353.5 ± 30.3 ^b^	154.3 ± 0.5 ^d^	Nd
Fresh black sesame	126.7 ± 4.2 ^c^	159.8 ± 1.3 ^b^	733.9 ± 14.8 ^b^	120.5 ± 0.1 ^a^	113.9 ± 0.3 ^b^	Nd
Roasted white sesame	79.3 ± 0.8 ^a^	167.0 ± 3.8 ^b^	442.7 ± 11.4 ^a^	294.0 ± 4.5 ^b^	133.6 ± 2.8 ^c^	Nd
Roasted black sesame	105.4 ± 1.3 ^b^	185.5 ± 4.4 ^c^	859.0 ± 16.6 ^c^	117.9 ± 2.4 ^a^	104.7 ± 1.4 ^a^	Nd

Results are presented as average values ± SD (*n* = 2). Values followed by identical superscript letters along the column are statistically similar. Nd stands for Not detected.

**Table 5:** TPC and antioxidant capacity values in different concentrations (50–200 mg/L) of pure sesamin and sesamolin standards.

Concentration of Sesamin and Sesamolin (mg/L)	TPC (mg GAE/L)		FRAP (mg TE/L)		CUPRAC (mg TE/L)	
	Sesamin	Sesamolin	Sesamin	Sesamolin	Sesamin	Sesamolin
50	9.0 ± 0.1	9.3 ± 0.1	0.3 ± 0.0	1.2 ± 0.1	9.4 ± 0.5	17.9 ± 0.5
100	10.2 ± 0.1	11.0 ± 0.1	0.7 ± 0.0	2.0 ± 0.1	10.1 ± 0.5	18.6 ± 0.5
150	10.6 ± 0.1	13.3 ± 0.1	1.3 ± 0.0	3.0 ± 0.1	12.9 ± 0.5	21.1 ± 1.0
200	12.2 ± 0.1	16.0 ± 0.1	1.6 ± 0.1	3.6 ± 0.1	15.1 ± 0.5	21.5 ± 0.5

Results are presented as average values ± SD (*n* = 2).

**Table 6:** TPC and antioxidant capacity values in different concentrations (10–70 mg/L) of pure sesamol standards.

Concentration of Sesamol (mg/L)	TPC (mg GAE/L)	FRAP (mg TE/L)	CUPRAC (mg TE/L)
10	17.3 ± 1.1	33.7 ± 2.5	44.0 ± 2.0
20	27.3 ± 1.0	71.1 ± 5.1	88.6 ± 4.5
50	46.3 ± 2.2	151.7 ± 13.6	160.1 ± 10.6
70	66.9 ± 3.3	226.5 ± 17.8	238.3 ± 18.2

Results are presented as average values ± SD (*n* = 2).

**Table 7:** TPC and antioxidant capacity in fresh white and black sesame oils extracted with a screw-press machine at different temperatures.

Screw-Press Temperature	TPC (mg GAE/100 g Oil DW)		FRAP (mg TE/100 g Oil DW)		CUPRAC (mg TE/100 g Oil DW)	
	White Sesame	Black Sesame	White Sesame	Black Sesame	White Sesame	Black Sesame
40 °C	44.7 ± 1.5 ^b^	54.6 ± 3.5 ^c^	25.1 ± 1.0 ^d^	28.4 ± 2.8 ^d^	90.8 ± 7.2 ^c^	217.6 ± 11.5 ^d^
50 °C	43.1 ± 1.1 ^b^	52.0 ± 5.0 ^c^	24.4 ± 1.2 ^cd^	27.7 ± 2.0 ^cd^	86.7 ± 9.2 ^bc^	213.9 ± 12.7 ^d^
60 °C	42.2 ± 1.8 ^b^	43.2 ± 1.9 ^b^	23.6 ± 1.0 ^cd^	27.2 ± 1.9 ^cd^	83.4 ± 6.6 ^abc^	199.0 ± 7.8 ^cd^
70 °C	40.4 ± 2.4 ^b^	41.4 ± 1.6 ^b^	23.2 ± 1.6 ^cd^	26.0 ± 2.5 ^cd^	81.4 ± 6.8 ^abc^	190.4 ± 8.5 ^cd^
80 °C	39.8 ± 1.6 ^b^	41.1 ± 2.3 ^b^	22.4 ± 0.7 ^cd^	24.9 ± 2.3 ^bcd^	81.1 ± 5.7 ^abc^	185.0 ± 7.0 ^c^
90 °C	39.5 ± 2.4 ^b^	40.2 ± 2.3 ^b^	21.9 ± 0.9 ^bcd^	23.5 ± 1.1 ^abcd^	79.8 ± 7.2 ^abc^	180.8 ± 12.3 ^bc^
100 °C	38.4 ± 4.1 ^b^	39.3 ± 4.1 ^b^	21.2 ± 1.2 ^abc^	22.9 ± 1.4 ^abc^	77.7 ± 1.2 ^abc^	172.7 ± 12.1 ^bc^
150 °C	25.3 ± 3.6 ^a^	29.1 ± 1.4 ^a^	19.0 ± 1.2 ^ab^	20.3 ± 1.2 ^ab^	69.0 ± 5.8 ^ab^	153.2 ± 9.3 ^ab^
200 °C	24.5 ± 2.8 ^a^	28.5 ± 0.9 ^a^	17.9 ± 1.3 ^a^	18.9 ± 1.3 ^a^	65.1 ± 6.6 ^a^	137.7 ± 4.7 ^a^

Results are presented as average values ± SD (*n* = 3). Values followed by identical superscript letters along the column are statistically similar.

**Table 8:** Lignan content in fresh white and black sesame oil extracted with a screw-press machine at different temperatures.

Screw-Press Temperature	Sesamin (mg/100 g Oil DW)		Sesamolin (mg/100 g Oil DW)		Sesamol (mg/100 g Oil DW)	
	White Sesame	Black Sesame	White Sesame	Black Sesame	White Sesame	Black Sesame
40 °C	456.7 ± 8.9 ^a^	182.2 ± 6.1 ^a^	218.8 ± 5.4 ^a^	188.6 ± 8.3 ^a^	7.5 ± 0.1 ^a^	9.5 ± 1.0 ^a^
50 °C	455.4 ± 6.2 ^a^	181.6 ± 8.6 ^a^	217.9 ± 1.1 ^a^	187.5 ± 6.8 ^a^	7.5 ± 0.0 ^a^	9.6 ± 0.7 ^a^
60 °C	453.4 ± 1.8 ^a^	180.2 ± 4.0 ^a^	216.8 ± 1.3 ^a^	185.6 ± 1.5 ^a^	7.6 ± 0.1 ^a^	9.6 ± 0.3 ^a^
70 °C	451.1 ± 3.2 ^a^	179.1 ± 2.5 ^a^	216.5 ± 2.6 ^a^	184.5 ± 1.7 ^a^	7.6 ± 0.1 ^ab^	9.6 ± 0.3 ^a^
80 °C	450.4 ± 4.5 ^a^	178.2 ± 2.7 ^a^	216.0 ± 1.6 ^a^	184.0 ± 2.9 ^a^	7.7 ± 0.0 ^ab^	9.7 ± 0.3 ^a^
90 °C	449.5 ± 2.2 ^a^	175.1 ± 4.7 ^a^	215.3 ± 1.0 ^a^	183.6 ± 4.9 ^a^	7.8 ± 0.1 ^ab^	9.7 ± 0.1 ^a^
100 °C	447.8 ± 10.2 ^a^	174.7 ± 5.1 ^a^	214.9 ± 3.5 ^a^	182.4 ± 4.9 ^a^	7.8 ± 0.2 ^ab^	9.8 ± 0.3 ^a^
150 °C	446.7 ± 1.4 ^a^	174.4 ± 2.9 ^a^	213.7 ± 2.7 ^a^	182.0 ± 3.4 ^a^	8.1 ± 0.2 ^b^	10.0 ± 0.1 ^a^
200 °C	446.3 ± 1.6 ^a^	173.2 ± 2.5 ^a^	212.9 ± 3.6 ^a^	181.3 ± 4.3 ^a^	9.3 ± 0.4 ^c^	10.3 ± 0.3 ^a^

Results are presented as average values ± SD (*n* = 3). Values followed by identical superscript letters along the column are statistically similar.

**Table 9:** Composition of fatty acids in the fresh sesame screw-pressed oil at different temperatures.

Fatty Acids	Fatty Acid (% of Total Fatty Acids) in Sesame Oil Extracted at Different Temperatures																	
	White	Black	White	Black	White	Black	White	Black	White	Black	White	Black	White	Black	White	Black	White	Black
Saturated Fatty Acids (SFAs)	40 °C	40 °C	50 °C	50 °C	60 °C	60 °C	70 °C	70 °C	80 °C	80 °C	90 °C	90 °C	100 °C	100 °C	150 °C	150 °C	200 °C	200 °C
Palmitic acid (C16:0)	9.3 ± 0.1	9.8 ± 0.1	8.7 ± 0.1	9.7 ± 0.1	8.9 ± 0.1	9.7 ± 0.1	8.7 ± 0.1	9.6 ± 0.1	8.7 ± 0.0	9.5 ± 0.1	8.6 ± 0.1	9.1 ± 0.4	8.6 ± 0.1	8.8 ± 0.1	8.6 ± 0.0	9.5 ± 0.0	8.6 ± 0.0	9.5 ± 0.0
Stearic acid (C18:0 *n*-9)	6.2 ± 0.0	6.3 ± 0.0	5.7 ± 0.1	6.4 ± 0.1	5.8 ± 0.0	6.3 ± 0.0	5.7 ± 0.1	6.4 ± 0.1	5.6 ± 0.0	6.4 ± 0.0	5.7 ± 0.0	6.1 ± 0.2	5.6 ± 0.1	6.0 ± 0.0	5.7 ± 0.1	6.4 ± 0.0	5.7 ± 0.1	6.4 ± 0.0
Total SFAs	15.5 ± 0.1	16.1 ± 0.1	14.4 ± 0.2	16.1 ± 0.2	14.6 ± 0.2	16.0 ± 0.1	14.4 ± 0.2	15.9 ± 0.2	14.3 ± 0.1	15.9 ± 0.2	14.3 ± 0.1	15.2 ± 0.6	14.2 ± 0.2	14.8 ± 0.2	14.3 ± 0.1	15.9 ± 0.1	14.3 ± 0.1	15.9 ± 0.1
Monounsaturated Fatty Acids (MUFAs)																		
Oleic acid (C18:1)	42.5 ± 0.1	42.0 ± 0.1	42.3 ± 0.1	42.1 ± 0.1	42.3 ± 0.1	42.2 ± 0.1	42.3 ± 0.2	42.2 ± 0.1	42.3 ± 0.2	42.2 ± 0.1	42.3 ± 0.2	42.5 ± 0.4	42.3 ± 0.2	42.8 ± 0.1	42.2 ± 0.1	42.2 ± 0.1	42.2 ± 0.1	42.2 ± 0.1
Eicosenoic acid (C20:1)	0.8 ± 0.0	0.9 ± 0.0	0.5 ± 0.0	0.8 ± 0.0	0.5 ± 0.0	0.9 ± 0.0	0.5 ± 0.0	0.9 ± 0.0	0.5 ± 0.0	0.9 ± 0.0	0.5 ± 0.0	0.7 ± 0.0	0.5 ± 0.0	0.6 ± 0.0	0.5 ± 0.0	0.7 ± 0.0	0.5 ± 0.0	0.7 ± 0.0
Total MUFAs	43.3 ± 0.2	42.9 ± 0.1	42.7 ± 0.1	42.9 ± 0.2	42.7 ± 0.1	43.0 ± 0.1	42.8 ± 0.2	43.1 ± 0.1	42.7 ± 0.2	43.1 ± 0.1	42.8 ± 0.3	43.2 ± 0.5	42.7 ± 0.2	43.4 ± 0.1	42.7 ± 0.2	42.9 ± 0.1	42.7 ± 0.2	42.9 ± 0.1
Polyunsaturated Fatty Acids (PUFAs)																		
Linoleic acid (C18:2 *n*-9, 12)	40.4 ± 0.0	40.5 ± 0.1	42.0 ± 0.1	40.5 ± 0.1	41.8 ± 0.1	40.5 ± 0.1	41.9 ± 0.1	40.5 ± 0.1	42.0 ± 0.2	40.6 ± 0.2	42.0 ± 0.1	40.8 ± 0.1	42.1 ± 0.4	40.9 ± 0.2	42.0 ± 0.1	40.5 ± 0.1	42.0 ± 0.1	40.5 ± 0.1
α-Linolenic acid (C18:3 *n*-9, 12, 15)	0.3 ± 0.0	0.3 ± 0.0	Nd	0.3 ± 0.0	Nd	0.3 ± 0.0	Nd	0.4 ± 0.0	Nd	0.4 ± 0.0	Nd	0.3 ± 0.0	Nd	0.3 ± 0.0	Nd	0.4 ± 0.0		0.4 ± 0.0
Total PUFAs	40.7 ± 0.0	40.8 ± 0.1	42.0 ± 0.1	40.8 ± 0.1	41.8 ± 0.1	40.8 ± 0.1	41.9 ± 0.1	40.8 ± 0.1	42.0 ± 0.2	40.9 ± 0.2	42.0 ± 0.1	41.1 ± 0.2	42.1 ± 0.4	41.2 ± 0.3	42.0 ± 0.1	40.8 ± 0.1	42.0 ± 0.1	40.8 ± 0.1
Total MUFAs + PUFAs	84.0 ± 0.2	83.7 ± 0.2	84.7 ± 0.3	83.7 ± 0.3	84.5 ± 0.2	83.9 ± 0.2	84.7 ± 0.3	83.9 ± 0.2	84.7 ± 0.3	84.0 ± 0.3	84.8 ± 0.3	84.3 ± 0.6	84.9 ± 0.6	84.6 ± 0.4	84.7 ± 0.3	83.7 ± 0.2	84.7 ± 0.3	83.7 ± 0.2
Ratio MUFAs + PUFAs/SFAs	5.4	5.2	5.9	5.2	5.8	5.2	5.9	5.3	5.9	5.3	5.9	5.6	6.0	5.7	5.9	5.3	5.9	5.3

Results are presented as average values ± SD (*n* = 3). Nd stands for Not detected.

**Table 10:** TPC and antioxidant capacity in roasted white and black sesame oils extracted with a screw-press machine at different temperatures.

Screw-Press Temperature	TPC (mg GAE/100 g Oil DW)		FRAP (mg TE/100 g Oil DW)		CUPRAC (mg TE/100 g Oil DW)	
	White Sesame	Black Sesame	White Sesame	Black Sesame	White Sesame	Black Sesame
40 °C	34.7 ± 1.3	43.6 ± 2.1	32.7 ± 1.9	39.4 ± 2.1	99.3 ± 2.5	242.4 ± 3.1
200 °C	18.9 ± 0.8	24.8 ± 1.1	21.2 ± 0.6	25.3 ± 1.2	71.1 ± 2.8	179.1 ± 2.9
*p*-value	*	*	*	*	*	*

Results are presented as average values ± SD (*n* = 3). The symbol “*” indicates statistical significance at *p* < 0.05.

**Table 11:** Lignan content in roasted white and black sesame oil extracted with a screw-press machine at different temperatures.

Screw-Press Temperature	Sesamin (mg/100 g Oil DW)		Sesamolin (mg/100 g Oil DW)		Sesamol (mg/100 g Oil DW)	
	White Sesame	Black Sesame	White Sesame	Black Sesame	White Sesame	Black Sesame
40 °C	438.4 ± 9.3	166.8 ± 8.3	208.9 ± 9.2	175.7 ± 8.8	22.1 ± 5.6	25.2 ± 1.6
200 °C	429.3 ± 13.8	151.0 ± 10.3	199.7 ± 10.7	168.7 ± 9.5	25.8 ± 3.1	27.7 ± 1.1
*p*-value	NS	NS	NS	NS	NS	NS

Results are presented as average values ± SD (*n* = 3). The symbol “NS” indicates no significant difference at *p* < 0.05.

**Table 12:** Composition of fatty acids in the roasted sesame screw-pressed oil at different temperatures.

Fatty Acids	Fatty Acid (% of Total Fatty Acids) in Sesame Oil Extracted at Different Temperatures			
	White	Black	White	Black
Saturated Fatty Acids (SFAs)	40 °C	40 °C	200 °C	200 °C
Palmitic acid (C16:0)	9.0 ± 0.1	9.6 ± 0.1	8.7 ± 0.1	9.4 ± 0.1
Stearic acid (C18:0 *n*-9)	5.9 ± 0.0	6.4 ± 0.0	5.5 ± 0.1	6.6 ± 0.1
Total SFAs	14.9 ± 0.1	16.0 ± 0.1	14.2 ± 0.2	16.0 ± 0.2
Monounsaturated Fatty Acids (MUFAs)				
Oleic acid (C18:1)	42.3 ± 0.1	42.2 ± 0.1	42.1 ± 0.1	42.3 ± 0.1
Eicosenoic acid (C20:1)	0.6 ± 0.0	0.8 ± 0.0	0.4 ± 0.0	0.8 ± 0.0
Total MUFAs	42.9 ± 0.2	43.0 ± 0.1	42.5 ± 0.1	43.1 ± 0.2
Polyunsaturated Fatty Acids (PUFAs)				
Linoleic acid (C18:2 *n*-9, 12)	40.2 ± 0.0	40.2 ± 0.1	41.7 ± 0.1	40.3 ± 0.1
α-Linolenic acid (C18:3 *n*-9, 12, 15)	0.3 ± 0.0	0.3 ± 0.0	Nd	0.2 ± 0.0
Total PUFAs	40.5 ± 0.0	40.5 ± 0.1	41.7 ± 0.1	40.5 ± 0.1
Total MUFAs + PUFAs	83.4 ± 0.2	83.5 ± 0.2	84.2 ± 0.3	83.6 ± 0.3
Ratio MUFAs + PUFAs/SFAs	5.6	5.2	5.9	5.2

Results are presented as average values ± SD (*n* = 3). Nd stands for Not detected.

## Data Availability

The original contributions presented in this study are included in the article. Further inquiries can be directed to the corresponding author.
